# WRKY70 and its homolog WRKY54 negatively modulate the cell wall-associated defenses to necrotrophic pathogens in Arabidopsis

**DOI:** 10.1371/journal.pone.0183731

**Published:** 2017-08-24

**Authors:** Jing Li, Rusen Zhong, E. Tapio Palva

**Affiliations:** 1 School of Chemistry, Chemical Engineering and Life Sciences, Wuhan University of Technology, Wuhan, China; 2 Viikki Biocenter, Department of Biosciences, Division of Genetics, University of Helsinki, Helsinki, Finland; Indiana University, UNITED STATES

## Abstract

Previous studies have identified the *Arabidopsis thaliana* transcription factor WRKY70 as a node of convergence for salicylic acid (SA) and jasmonic acid (JA)-mediated defense signal pathways and, together with its closest homolog WRKY54, as a negative regulator of SA biosynthesis. Here, we demonstrate that WRKY70 together with WRKY54 negatively affect the response of Arabidopsis to the necrotrophic pathogens *Pectobacterium carotovorum* and *Botrytis cinerea*, but not to the hemibiotroph *Pseudomonas syringae* pv *tomato* (*Pst*) DC3000, as revealed by mutants studies. Unstressed *wrky54wrky70* double mutants exhibited increased levels of SA, accumulation of hydrogen peroxide (H_2_O_2_) and up-regulated expression of both SA and JA/ethylene (ET) responsive defense related genes. Additionally, protein cross-linking in cell wall was promoted by endogenous SA, suggesting involvement of wall-associated defenses against necrotrophs. This response to necrotrophs was compromised by introducing the *sid2-1* allele impairing SA biosynthesis and leading to reduction of H_2_O_2_ content and of defense gene expression. The data suggest that the elevated SA level in the *wrky54wrky70* double mutant results in moderate accumulation of H_2_O_2_, in promoting cell wall fortification and consequently enhanced resistance to necrotrophs but is not sufficient to trigger hypersensitive reaction (HR)-like cell death and resistance to biotrophs/hemibiotrophs like *Pst* DC3000.

## Introduction

In nature, plants are always surrounded by a series of potentially pathogenic microorganisms. According to the way in which pathogens derive their nutrients, they can be divided into two major classes [1–4]: biotrophs, which suppress plant defense responses by secretion of effectors and gain their nutrients from living host tissues [1, 3], and necrotrophs, which kill the host tissues and feed on dead cells by employing plant cell wall-degrading enzymes (CWDEs), necrosis-inducing proteins and toxins [1–4]. Some pathogens, hemibiotrophs, may behave differently under different environmental conditions or stages of their life cycles e.g. with a biotrophic phase in the beginning of infection followed by a necrotrophic phase when the infection is fully established [1, 3]. Regardless of the type of attacking pathogen, plants employ a variety of defense strategies to protect themselves, including both preformed mechanisms and infection-induced responses. These responses include the strengthening of plant cuticle or cell wall through production of callose, lignin or suberin, production of antimicrobial proteins and metabolites, such as pathogenesis-related proteins and phytoalexins as well as reactive oxygen species (ROS) and hormones [1, 2, 5]. Three well known phytohormones, salicylic acid (SA), jasmonic acid (JA) and ethylene (ET) are central in regulation of different signaling pathways in plant defense to distinct pathogens. The SA-mediated signaling pathway is generally associated with biotroph resistance, while JA/ET dependent signaling pathways are involved in defense to necrotrophs [1, 5–7]. Pathogen response involves induction of hormone-specific target genes encoding antimicrobial proteins, such as genes for PATHOGENESIS-RELATED PROTEIN 1 (PR1), BETA 1, 3-GLUCANASE (PR2) and THAUMATIN-LIKE PROTEIN (PR5) by SA, followed by the establishment of systemic acquired resistance (SAR), whereas genes encoding PLANT DEFENSIN 1.2 (PDF1.2), BASIC CHITINASE (PR3) and HEVEIN-LIKE (PR4) are induced by JA/ET in *Arabidopsis thaliana* [5–7]. These two distinct branches of hormone-mediated defense signaling are often antagonistic [5–9], although even synergistic interactions between SA and JA/ET dependent pathways have been implicated [7, 10–12].

In addition to the hormone-mediated immune responses, accumulation of ROS, such as hydrogen peroxide (H_2_O_2_) or superoxide (O_2_^-^) can trigger papilla formation and assembly of natural barriers in the basal defense. Likewise, different types of ROS are also involved in the activation of defense responses [2, 13–16]. In SAR, SA and ROS play synergistic roles in a signal amplification loop to drive the hypersensitive response (HR) associated cell death. This constrains the growth of biotrophic pathogens at the site of infection, but does not necessarily limit the infection of necrotrophic pathogens but may rather facilitate their growth [2, 13]. Although the HR-induced cell death could promote infection by necrotrophic pathogens, the early accumulation of moderate levels of ROS is believed to be beneficial to plant resistance also to necrotrophs, triggering induction of secondary metabolites, defense signals, antimicrobial compounds and reinforcement of cell wall [17]. For example, a specific set of genes involved in SA- and JA/ET-mediated defense signaling were up-regulated in plants exposed to exogenous ROS [17–19]. Thus, the role of ROS in plant defense can be either beneficial or harmful: depending on the actual cellular levels. It can efficiently influence both SA-dependent SAR against biotrophs and JA/ET-mediated resistance restricting the growth of necrotrophic pathogens [13, 17, 19, 20].

Previous studies have shown that the WRKY transcription factor WRKY70 acts as a node of convergence for integrating signals from SA and JA-dependent defense pathways in Arabidopsis [8, 21]. Overexpression of *WRKY70* was shown to promote up-regulation of SAR-related defense genes and resistance to the hemibiotroph *Pseudomonas syringae* pv *tomato* DC3000 (*Pst* DC3000) and the biotroph *Erysiphe cichoracearum* while enhancing susceptibility to the necrotroph *Alternaria brassicicola* [8, 21]. Furthermore, Wang [22] showed that WRKY70 and its closest homolog WRKY54 cooperated as negative regulators of SA biosynthesis. Intriguingly, while the *wrky54wrky70* double mutant exhibited significantly increased levels of SA, the resistance to *Pseudomonas syringae* pv. *maculicola* (*Psm*) ES4326 was not enhanced [22]. To explore the role of WRKY70 and WRKY54 in plant defense to different classes of pathogens, we characterized the effect of single and double mutants of *WRKY70* and *WRKY54* on resistance to the necrotrophs *Pectobacterium carotovorum* and *Botrytis cinerea* as well as to the hemibiotroph *Pst* DC3000. Intriguingly, resistance to *P*. *carotovorum* can be induced both by SA- and JA/ET-mediated defenses [23]. Our current study demonstrates that loss of both *WRKY54* and *WRKY70* clearly enhanced the resistance of Arabidopsis to necrotrophic bacterial and fungal pathogens *P*. *carotovorum* and *B*. *cinerea*, respectively. The elevated SA level in the *wrky54wrky70* double mutant led to moderate accumulation of ROS, resulting in up-regulation of defense related genes and activation of cell wall-associated defenses. In contrast, the resistance to the hemibiotroph *Pst* DC3000 in the *wrky54wrky70* double mutant was not enhanced although most SA-induced genes were up-regulated, suggesting that additional WRKY54 and WRKY70 controlled processes could be necessary for development of resistance to biotrophs.

## Materials and methods

### Plant material and growth conditions

Arabidopsis were germinated and transferred to the soil in a chamber at 22°C with 70/90% relative humidity and under a light/dark cycle of 12/12 h. The plants used for experiments were grown for 3 or 4 weeks at vegetative stage (before bolting). All the genotypes including wild type, *wrky54*, *wrky70*, and *sid2-1* single (the *sid2-1* mutant lacks an enzyme required for SA biosynthesis), *wrky54wrky70* double, *wrky54wrky70sid2-1* triple mutants as well as *WRKY70* overexpressor used in this study have been described in Li *et al*. [24].

### Microarray data analysis

Microarray data processing and analysis were described previously [24]. The raw data are available in GEO with accession number GSE38522.

### Quantitative RT-PCR

The RNA was isolated according to Besseau *et al*. [25]. The methods used for qRT-PCR were described in Li *et al*. [24]. The primers used in this study are listed in S3 Table, with *ACTIN2* (At3g18780) used as reference gene.

### Protein extraction and western blot analysis

Total protein was extracted from 100mg tissue of 3-week-old plants under non-treated conditions. The powder ground in liquid nitrogen was mixed with 200μl protein extraction buffer (50 mM Tris-HCl pH = 7.5, 150mM NaCl, 1mM EDTA, 10% glycerol, 1mM DTT, 1mM Pefablock SC (Roche), 1×Complete Protease Inhibitor Cocktail (Roche), 1% Triton X-100). The homogenized solution was incubated on ice for 30 min. Insoluble material was spinned down at 4°C for 10 min at 12000×g. The supernatant was collected to a new eppendorf tube and the protein concentration was determined by Bio-Rad assay.

Twenty μg of total protein was resolved by Any kD^™^ Mini-PROTEAN^®^ TGX^™^ Precast Gel (Bio-Rad) and transferred to nitrocellulose membranes (GE Healthcare) by semi-dry blotting. Blot was incubated in the primary antibody of PR2 (pathogenesis-related protein 2) (Agrisera) at a dilution of 1:2500 for 1h at room temperature with agitation, followed by washing with TBS-T for 3 times. Then the blot was incubated in the secondary antibody, HRP-conjugated Anti-rabbit IgG (Cell Signaling Technology) at a dilution of 1:5000 for 1h at room temperature with agitation. The membrane was washed again as above and developed with ECL prime western blotting detection reagent (GE Healthcare), and the signals were detected with ECL Hyperfilm (GE Healthcare) after 10 min of exposure.

### Pathogen infections

Inoculation with *Pectobacterium carotovorum* subsp *carotovorum* SCC1 was done either by pipetting 10μl of the bacterial suspension (10^6^ cells/ml in 50mM NaCl) on the leaves of 3 or 4 weeks old plants or spraying the whole plants (10^8^ cells/ml in 50mM NaCl, and 60 ml for 72 plants in one tray). The lesion diameter measurement, bacterial titering as well as qRT-PCR for gene expression were performed 24h after the infection. *Botrytis cinerea*, strain B05.10 was used to the fungal infection experiments. Each leaf was infected by 5μl of spore suspension with the concentration of 2×10^6^ ml^-1^ or the whole plants were sprayed with the same spore suspension (60 ml for 72 plants in one tray). The qRT-PCR for gene expression and lesion diameters measurements were monitored after 24 and 48h, respectively. For *Pseudomonas syringae* pv *tomato* DC3000, plants were sprayed by suspension of 10^6^ cells/ml in 10mM MgCl_2_ (60 ml for 72 plants in one tray). Sprayed leaves were harvested 4d after infection for bacterial titering. For cell wall fortification test, the supernatant of overnight culture of *P*. *carotovorum* was diluted 1:1 and 1μl of diluted solution was pipetted onto the leaves of 3 weeks old seedlings for each line [4]. The phenotypes were photographed 6h after inoculation.

### Diaminobenzidine (DAB), trypan blue and coomassie blue staining

To visualize the reactive oxygen species (ROS) production, rosette leaves were inoculated with mock and bacterial suspension and stained after 6h with 3,3’-diaminobenzidine (DAB). The leaves were vacuum infiltrated with 0.1% DAB in 10mM MES, pH 6.5 for 30 min, followed by clearing in boiling alcohol-lactophenol (2:1) for 5 min. The leaves were rinsed 2 times with 50% ethanol. To visualize the cell death, the leaves were collected 48h after spraying with fungal spore suspension and boiled in trypan blue solution for 3 min. Then the leaves were transferred to chloral hydrate solution for destaining. For protein cross-linking, three-week-old plants were sprayed with 5mM SA, the leaves were collected after 6 h and 24 h. Non-treated plants were used as control. Ethanol-fixed samples were placed in 1% SDS at 80°C for 24h and stained with 0.1% coomassie blue in 40% ethanol/10% acetic acid for 15 min. Then the leaves were washed in 40% ethanol/10% acetic acid. The Microscopy was performed with a Leitz Laborlux S microscope (Leica, Wetzlar, Germany).

### Assay for callose deposition

Three weeks old in vitro Arabidopsis plants were sprayed with 100μg/ml oligogalacturonide (OG) solution and kept at high humidity for 24h. At least three leaves from independent plants of each line were harvested and placed in sterile 12-well plates. The detection of callose was performed using aniline blue staining as described in Daudi *et al*. [26].

## Results

### Defense related genes are up-regulated in the *wrky54wrky70* double mutant

Previously, we have explored the involvement of WRKY54 and WRKY70 in osmotic stress response in *Arabidopsis* [24]. Analysis of the microarray data also indicated drastic alterations in expression of defense related genes in the *wrky54wrky70* double mutant relative to the wild type under non-induced conditions. As shown in S1 Table, over 700 probes showed significant up-regulation in the *wrky54wrky70* double mutant compared to wild type under control conditions (log_2_FC≥1.5). GO annotation analysis highlighted 89 significant GO terms (S2 Table), the majority of the GO categories could be assigned to defense response to stimulus. The defense response GO class 0006952 contained 73 probes, from which we selected representative defense-related marker genes including SA-inducible *PR* genes, JA/ET-inducible *PDF* genes as well as H_2_O_2_ responsive genes (Table 1).

**Table 1:** Defense-related genes up-regulated in the non-treated *wrky54wrky70* double mutant.

AGILENT_ID	Description	*wrky54wrky70*_ctrl VS Col-WT_ctrl (log_2_FC)
A_84_P17268	Arabidopsis thaliana PR1 (PATHOGENESIS-RELATED GENE 1)	6.63
A_84_P14560	Arabidopsis thaliana PAD3 (PHYTOALEXIN DEFICIENT 3)	5.63
A_84_P22787	Arabidopsis thaliana ATGSTF7 (GLUTATHIONE S-TRANSFERASE 11)	5.21
A_84_P196694	Arabidopsis thaliana PDF1,2c (plant defensin 1,2c)	4.93
A_84_P239215	Arabidopsis thaliana PDF1,3 (plant defensin 1,3)	4.90
A_84_P137009	Arabidopsis thaliana PDF1,2 (Low-molecular-weight cysteine-rich 77)	4.73
A_84_P23050	Arabidopsis thaliana PR4 (PATHOGENESIS-RELATED 4)	4.19
A_84_P16574	Arabidopsis thaliana BGL2 (PATHOGENESIS-RELATED PROTEIN 2) (PR2)	3.63
A_84_P589894	Arabidopsis thaliana ICS1 (ISOCHORISMATE SYNTHASEI)	3.59
A_84_P844839	Arabidopsis thaliana EDS5 (ENHANCED DISEASE SUSCEPTIBILITY 5)	3.35
A_84_P118682	Arabidopsis thaliana PAD4 (PHYTOALEXIN DEFICIENT 4)	3.12
A_84_P14299	Arabidopsis thaliana PR5 (PATHOGENESIS-RELATED GENE 5)	3.00
A_84_P20293	Arabidopsis thaliana ATHCHIB (BASIC CHITINASE) (PR3)	2.65
A_84_P812454	Arabidopsis thaliana ATGSTF6 (EARLY RESPONSIVE TO DEHYDRATION 11) (GST1)	2.42

**Fig 1:**
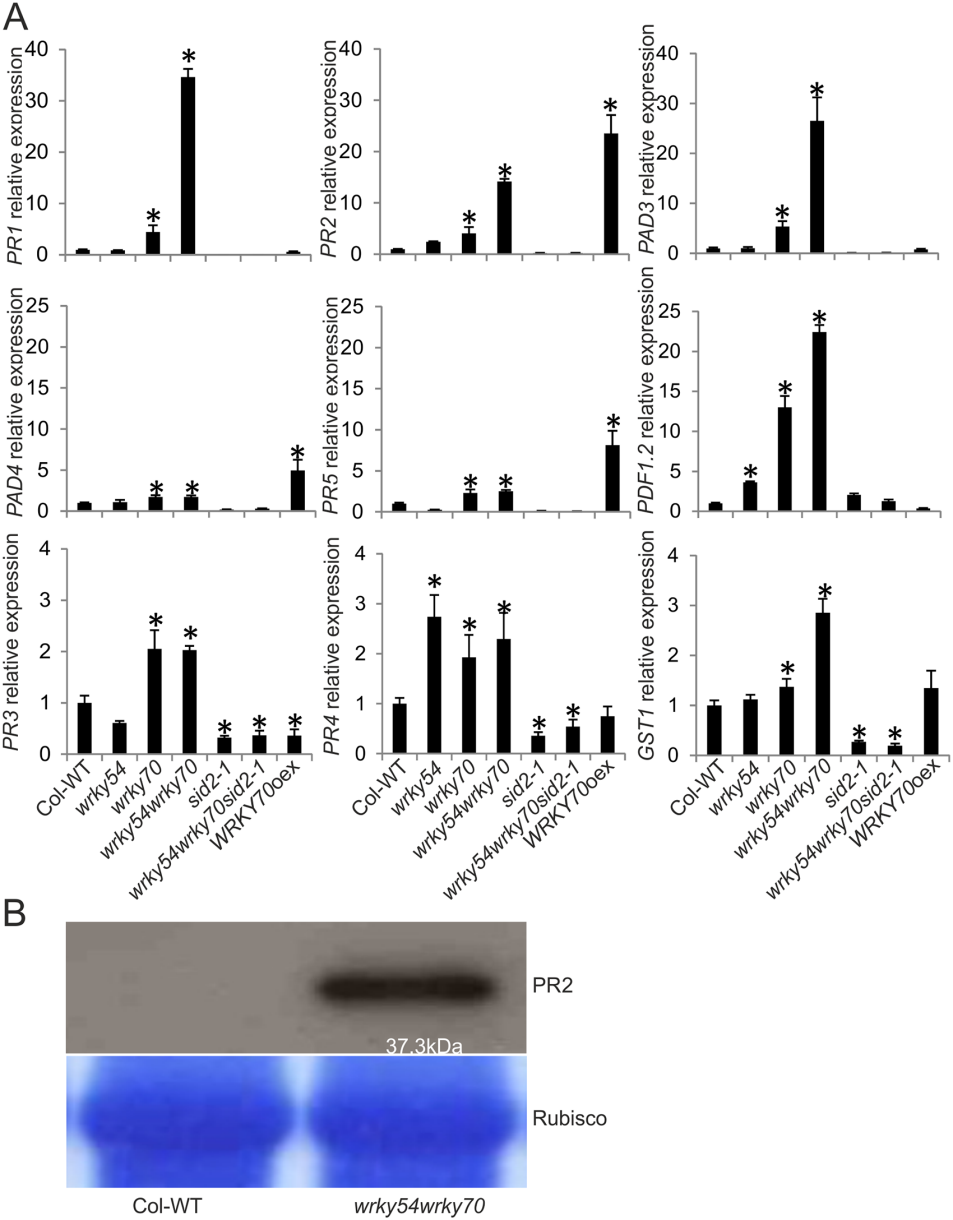
Expression of defense marker genes in non-treated Arabidopsis wild type (Col-WT), *wrky54*, *wrky70*, *sid2-1* single, *wrky54wrky70* double, *wrky54wrky70sid2-1* triple mutants, as well as *WRKY70* overexpressor line (*WRKY70*oex). (A) Leaves from untreated three-week-old Arabidopsis plants were collected and RNA was extracted for cDNA synthesis. Gene expression was assayed by quantitative reverse transcription-polymerase chain reaction (qRT-PCR). The relative expression of each marker gene was normalized to that of ACT2. Values were obtained from the means ± SD of three technical replicates (*, P<0.01, one-way ANOVA test). Three independent assays were performed with similar results. (B) Western blot analysis of total protein extracted from the leaves of three-week-old plants without treatment. Total protein (20μg) was used for the separation on Any kD^™^ Mini-PROTEAN^®^ TGX^™^ Precast Gel (Bio-Rad). The protein molecular mass for beta 1,3-glucanase (PR2) is indicated under the band in the upper panel. Rubisco stained with PageBlue was used as a control for equal loading of proteins.

To confirm the results from the microarray analysis, the expression levels of the marker genes were characterized in different genotypes (wild type, *wrky54*, *wrky70*, and *sid2-1* single, *wrky54wrky70* double, *wrky54wrky70sid2-1* triple mutants as well as *WRKY70* overexpressor) under non-induced conditions by quantitative reverse transcription-polymerase chain reaction (qRT-PCR) (Fig 1A). The tested genes included *PR1*, *PR2*, *PR5* and *PAD4* (*PHYTOALEXIN DEFICIENT 4*), considered as markers for the SA-mediated responses [5]; *PDF1*.*2*, *PR3*, *PR4* and *PAD3* (*PHYTOALEXIN DEFICIENT 3*) assigned as markers for JA/ET-mediated responses [5]; as well as *GST1* (*GLUTATHIONE TRANSFERASE 1*) induced by H_2_O_2_ [27, 28]. As shown in Fig 1A, all the tested genes were clearly up-regulated in the *wrky54wrky70* double mutant, although this up-regulation was not limited to the double mutant but was found in at least one of the other lines. In contrast, the basal expression levels of these genes except for *PDF1*.*2* were clearly reduced in the *sid2-1* single and *wrky54wrky70sid2-1* triple mutants (Fig 1A and S1 Table). These results suggest that the introduction of the SA-biosynthesis deficient *sid2-1* allele into *wrky54wrky70* results in reduced expression of different types of defense-related genes. Moreover, the increased expression of the JA/ET regulated genes in the *wrky70* single mutant was consistent with the previous conclusion that WRKY70 is a repressor of JA/ET responsive genes such as *PDF1*.*2* [21]. Our results also suggest that the loss of *WRKY70* could contribute to the enhanced basal expression of JA/ET responsive genes in the *wrky54wrky70* double mutant. The overexpressor of *WRKY70* showed elevated levels of transcripts of *PR* genes associated with SAR (*PR2*, *PR5*, *PAD4*), in agreement with Li *et al*. [8]. Furthermore, the expression of *GST1* was enhanced in *wrky54wrky70* double mutant under the non-stressed conditions, suggesting accumulation of H_2_O_2_ in the *wrky54wrky70* double mutant.

In accordance to the qRT-PCR results, a western blot analysis showed that corresponding defense related proteins were more abundant in the *wrky54wrky70* double mutant. This analysis was done under non-induced conditions using a PR2 specific antibody. As shown in Fig 1B, the band of PR2 was clearly visualized in *wrky54wrky70* double mutant, whereas no band was detected in wild-type plants. This supports the idea that both the defense-related transcripts and corresponding proteins such as PR2 are accumulated in the non-stressed *wrky54wrky70* double mutant.

### The *wrky54wrky70* double mutant is more resistant to a necrotrophic bacterial pathogen

**Fig 2:**
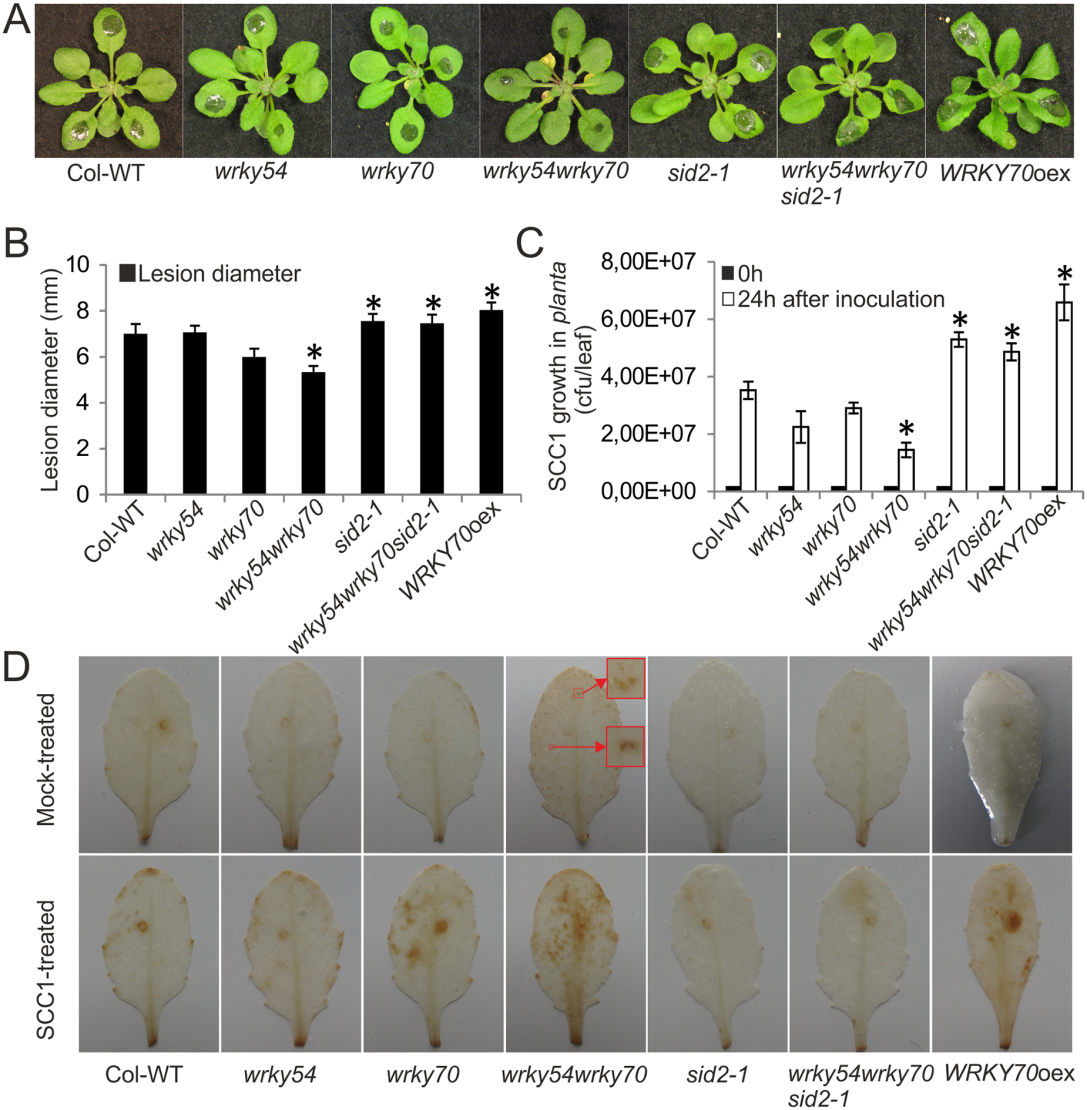
Enhanced resistance of the *wrky54wrky70* double mutant to the necrotrophic bacterium *Pectobacterium carotovorum* SCC1. (A) Three-week-old Arabidopsis plants were inoculated locally with *P*. *carotovorum* SCC1 (pipetting the bacterial solution to three leaves of each plant). Seventy-two plants of each line in one tray were used in one independent experiment. Representative plants were photographed 24h after infection. Three independent experiments were performed with similar results. (B) The disease symptoms were monitored after inoculation by measuring the average lesion diameters on three leaves of eight plants each. Values were mean ± SD of three independent experiments (*, P<0.01, one-way ANOVA test). (C) The growth of *P*. *carotovorum* SCC1 was evaluated 24h after inoculation in each line (pipetting the bacterial solution to three leaves of each plant). Colony-forming units of five plants for each line were determined. Error bars indicated ± SD of five biological replicates for each line. Three independent experiments with similar results were performed (*, P<0.01, one-way ANOVA test). (D) Three inoculated leaves of five plants each from four weeks old plants were stained with 3,3’-diaminobenzidine (DAB) 6h after mock and *P*. *carotovorum* SCC1 treatment to detect H_2_O_2_ accumulation. The boxed areas on the leaf indicate spots of accumulation of H_2_O_2_ in the mock-treated *wrky54wrky70* double mutant, and the corresponding magnified areas are pointed by the red arrows. At least three independent experiments were performed with similar results.

Prompted by the constitutive up-regulation of defense related genes in the *wrky54wrky70* double mutant, we examined the resistance of the double mutant to the bacterial necrotroph *P*. *carotovorum* SCC1. Loss of *WRKY54* and *WRKY70* enhanced the resistance of the plants to *P*. *carotovorum* infection, limiting the spreading of bacterial maceration in the *wrky54wrky70* double mutant (Fig 2A). Lesion diameter and bacterial growth were also measured to quantify the maceration phenotypes of the plants (Fig 2B and 2C). As shown in these results, wild-type plants and the *wrky54* single mutant showed increased lesion diameter and enhanced bacterial growth than those in the *wrky54wrky70* double mutant, whereas the *wrky70* single mutant displayed an intermediate phenotype with less symptoms than wild type and the *wrky54* single mutant, but not as resistant as the *wrky54wrky70* double mutant (Fig 2A–2C). In comparison, the *sid2-1* single and the *wrky54wrky70sid2-1* triple mutants as well as the overexpressor of *WRKY70* appeared to be even more susceptible than the wild type, showing more extensive expansion of the lesions and enhanced bacterial growth (Fig 2A–2C).

WRKY54 and WRKY70 have been identified as negative regulators of SA biosynthesis, acting through a negative feedback loop [22]. Consequently, the *wrky54wrky70* double mutant exhibits up-regulation of the SA biosynthesis-related *ICS1* gene (Table 1) resulting in a high constitutive SA level [22, 24]. SA has been shown to trigger an increase in endogenous ROS, such as H_2_O_2_, which in turn is proposed to be the signal leading to the defense response [20, 29, 30]. Accordingly, the high level of SA accumulated in the *wrky54wrky70* double mutant [24] was accompanied by a corresponding increase in the basal expression of *GST1* (Fig 1A). Consequently, we hypothesized that the H_2_O_2_ level would be higher in the *wrky54wrky70* double mutant than that normally found in the wild type. To determine if this was the case, leaves of each line either mock treated or inoculated by *P*. *carotovorum* were stained with 3,3’-diaminobenzidine (DAB), a histochemical reagent that detects H_2_O_2_ (Fig 2D). Very little brown precipitates indicative of H_2_O_2_ accumulation were visualized by DAB staining in mock treated leaves of the lines tested except for the *wrky54wrky70* double mutant, which exhibited clear brown spots throughout the whole leaf (Fig 2D). Six hours after *P*. *carotovorum* inoculation, the scattered H_2_O_2_ accumulation was much stronger and more evident in the *wrky54wrky70* double mutant, whereas in the wild type and the *wrky54* single mutant H_2_O_2_ accumulation was only slightly increased around the site of infection. The pattern of H_2_O_2_ accumulation in the *wrky70* single mutant appeared similar to that in the *wrky54wrky70* double mutant although less pronounced. The overexpressor of *WRKY70* showed enhanced H_2_O_2_ accumulation mainly localized at the site of inoculation. Interestingly, hardly any difference in H_2_O_2_ accumulation was detected in *sid2-1* single and *wrky54wrky70sid2-1* triple mutants when the mock treated and inoculated plants were compared (Fig 2D). In conclusion, the elevated SA level in the *wrky54wrky70* double mutant [24] might result in the accumulation of H_2_O_2_, and possibly contribute to the observed resistance to *P*. *carotovorum*.

### The resistance to a fungal necrotroph is enhanced in the *wrky54wrky70* double mutant

**Fig 3:**
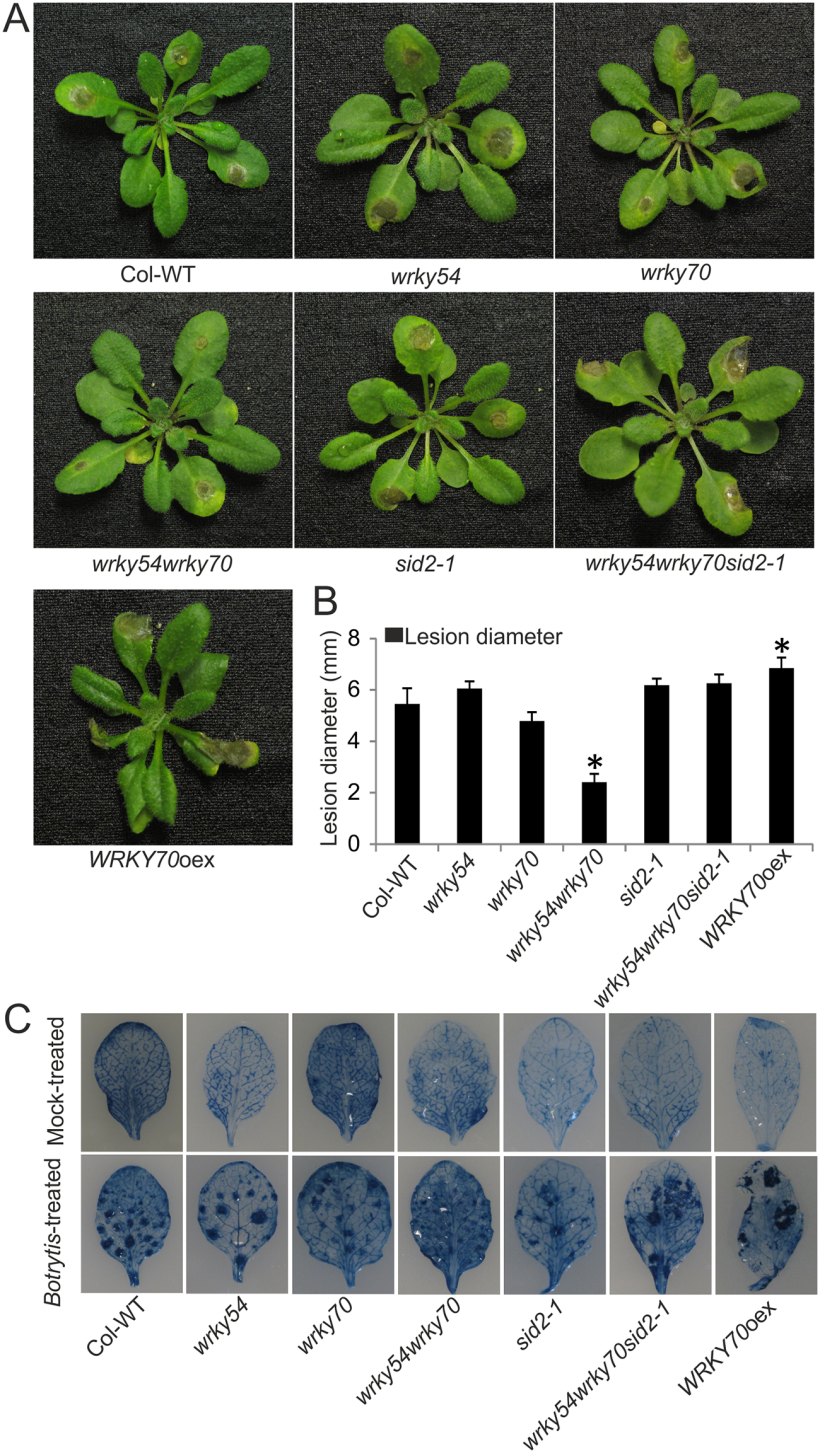
Enhanced resistance of the *wrky54wrky70* double mutant to the necrotrophic fungus *Botrytis cinerea* strain B05.10. (A) Three-week-old Arabidopsis plants were inoculated with *B*. *cinerea* strain B05.10 (pipetting the spore suspension to three leaves of each plant). Seventy-two plants of each line in one tray were used in one independent experiment. The representative plants were photographed 48h after inoculation. Three independent experiments were performed with similar results. (B) The disease symptom was evaluated by measuring the average lesion diameters on three leaves of eight plants each. Values were mean ± SD of three independent experiments (*, P<0.01, one-way ANOVA test). (C) Three spraying-inoculated leaves of five plants each from three-week-old plants were stained with trypan blue 48h after infection to visualize the cell death. Three independent experiments were performed with similar results.

To explore whether the enhanced resistance observed in the *wrky54wrky70* double mutant was specific to bacterial necrotrophs or of more general nature, we inoculated the *wrky54wrky70* double mutant as well as the other lines with the necrotrophic fungus *B*. *cinerea*. The disease phenotypes were recorded 48h after the inoculation by lesion diameter measurements (Fig 3A and 3B). In accordance with the results observed in response to inoculation with *P*. *carotovorum*, the maceration at the site of infection was drastically reduced in the leaves of the *wrky54wrky70* double mutant. In wild type, *wrky54* and *wrky70* single mutants, the lesions showed more extensive expansion than those in the double mutant, although the *wrky70* single mutant exhibited an intermediate phenotype. The enhanced resistance of the double mutant was compromised by introduction of the *sid2-1* allele. Moreover, the *WRKY70* overexpressor line also showed somewhat enhanced susceptibility to *B*. *cinerea*. (Fig 3A and 3B).

The necrotrophic fungal pathogen *B*. *cinerea* usually triggers plant cell death during early infection, which promotes successful colonization of the host by the fungus [2, 31]. To investigate the host cell death after infection by *B*.*cinerea*, leaves of each line were stained by trypan blue to reveal the dead cells (Fig 3C). In the mock-treated plant leaves, no visible cell death was detected in any of the genotypes. Even the mock-treated *wrky54wrky70* double mutant accumulating H_2_O_2_ (Fig 2D), did not show any cell death symptoms before infection. In contrast, cell death was induced in each line 48h post-inoculation by *B*.*cinerea* (Fig 3C). There was, however, a marked difference in the cell death phenotype of the *wrky54wrky70* double mutant: the development of cell death symptoms was much weaker compared to the other lines. This phenotype was abolished by introduction of the *sid2-1* allele (Fig 3C). Furthermore, the overexpressor of *WRKY70* presented considerable susceptibility to *B*.*cinerea* revealed by a large area of dead cells stained by trypan blue and also suggested that the enhanced cell death could promote the growth of *B*. *cinerea* in the *WRKY70* overexpressor (Fig 3).

### Resistance to the hemibiotroph *Pst* DC3000 is not enhanced in the *wrky54wrky70* double mutant

**Fig 4:**
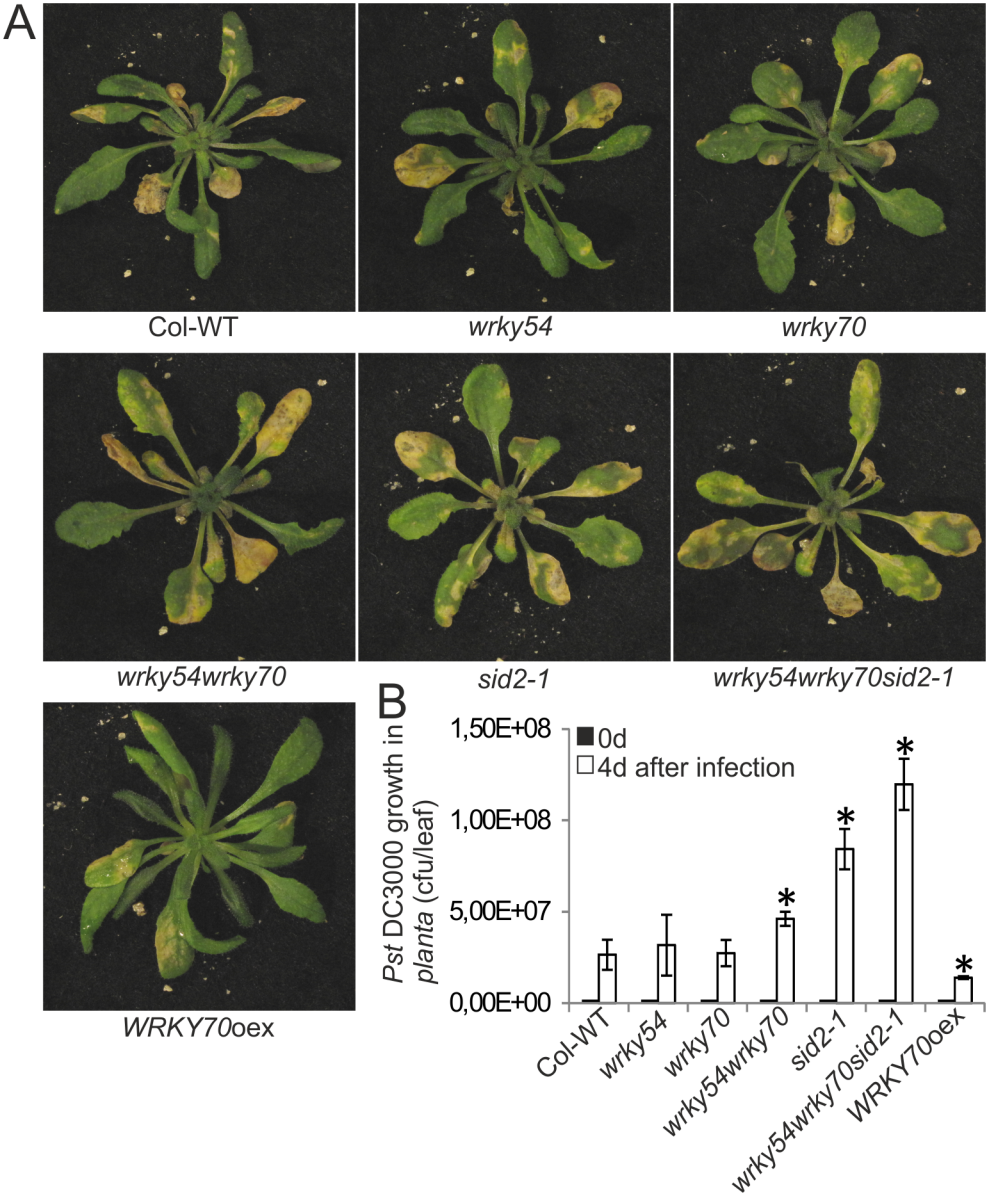
Resistance to the bacterial hemibiotroph *Pseudomonas syringae* pv *tomato* DC3000 (*Pst* DC3000) was not enhanced in the *wrky54wrky70* double mutant. (A) Three-week-old Arabidopsis plants were sprayed with *Pst* DC3000. Seventy-two plants of each line in one tray were used in one independent experiment. Representative plants were photographed 4d after infection. Three independent experiments were performed with similar results. (B) The growth of *Pst* DC3000 was determined 4d after infection in each line. Colony-forming units of five plants for each line were evaluated. Error bars indicated ± SD of five biological replicates for each line (*, P<0.01, one-way ANOVA test). Three independent experiments with similar results were performed.

Based on enhanced resistance of the *wrky54wrky70* double mutant to necrotrophs (Figs 2 and 3) and up-regulated expression of the SA-inducible *PR1*, *PR2*, *PR5* and *PAD4* genes (Fig 1), we expected that the mutant might also show resistance to the hemibiotroph *Pst* DC3000. To our surprise, there was however no increase in resistance of the *wrky54wrky70* double mutant to this pathogen. In contrast, bacterial growth in the *wrky54wrky70* double mutant was slightly enhanced compared to that observed in wild-type plants, and the corresponding single mutants (Fig 4). Moreover, the susceptibility of the *wrky54wrky70* double mutant was further increased by introduction of the *sid2-1* allele impaired in SA biosynthesis (Fig 4). This indicated that reduced SA level in *wrky54wrky70sid2-1* triple mutant further contributed to the increased susceptibility of the plants to *Pst* DC3000. Conversely, the *WRKY70* overexpressor line showed clearly enhanced resistance to *Pst* DC3000 (Fig 4), in accordance with the previous findings that WRKY70 plays a positive role in plant defense to biotrophs [8, 21].

We also characterized induced deposition of callose as an indicator of plant immunity [32, 33] in the different plant lines. As shown in S1 Fig, OG treatment caused a noticeable increase in callose deposition when compared to control plants. There was no significant difference in callose deposition between OG-treated wild type, *wrky54*, *wrky70* single and *wrky54wrky70* double mutants, whereas *sid2-1* single and *wrky54wrky70sid2-1* triple mutants showed clearly reduced amount of callose. In contrast, the OG-treated *WRKY70* overexpressor line showed increased deposition of callose. This is consistent with that the *WRKY70* overexpressor line is more resistant to *Pst* DC3000 (Fig 4). In conclusion, it appears that both WRKY54/70 controlled processes and SA are required for plant resistance to a hemibiotrophic pathogen.

### The resistance of the *wrky54wrky70* double mutant to necrotrophs involves enhanced expression of JA/ET responsive genes and cell wall-associated defenses

**Fig 5:**
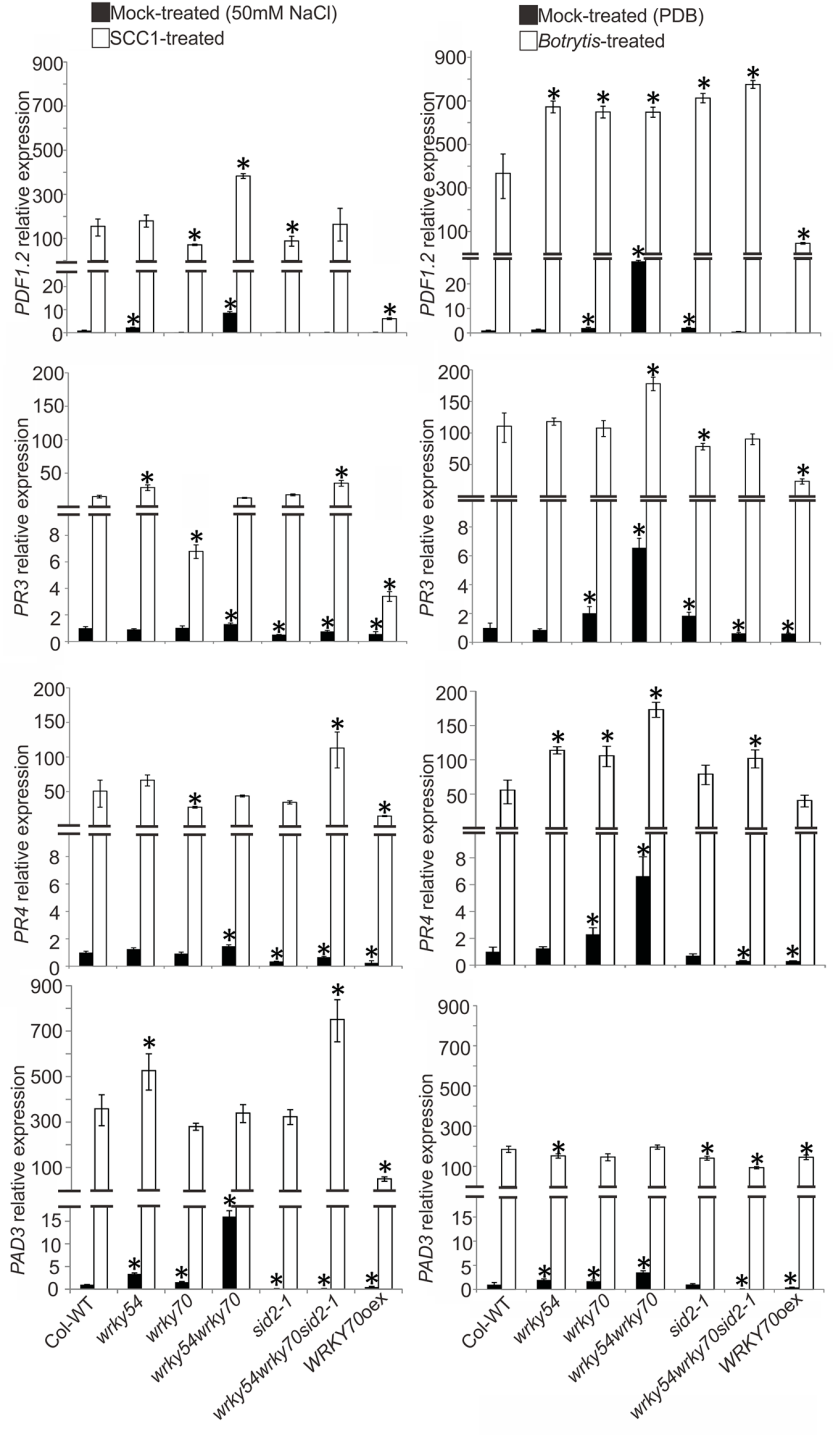
Expression of jasmonic acid responsive genes in Arabidopsis wild type (Col-WT), *wrky54*, *wrky70*, *sid2-1* single, *wrky54wrky70* double, *wrky54wrky70sid2-1* triple mutants, as well as *WRKY70* overexpressor line (*WRKY70*oex), 24h after spraying with *Pectobacterium carotovorum* or *Botrytis cinerea*. Leaves from three-week-old plants were collected for RNA extraction and cDNA synthesis, followed by quantitative reverse transcription-polymerase chain reaction (qRT-PCR) assay. The relative expression of each marker gene was normalized to that of *ACT2*. Values were obtained from the means ± SD of three technical replicates (*, P<0.01, one-way ANOVA test). Three independent experiments were performed with the similar results. PDB indicates potato dextrose broth.

JA/ET mediated responses including up-regulation of genes such as *PDF1*.*2*, *PR3*, *PR4*, *PAD3* are well known to be involved in resistance to necrotrophs [5]. To elucidate their contribution to the observed resistance phenotypes, we characterized the expression profiles of these four genes by qRT-PCR in the *wrky54wrky70* double mutant, along with other genotypes in response to the necrotrophs *P*. *carotovorum* and *B*. *cinerea* (Fig 5). The tested genes were up-regulated in mock-treated samples of the *wrky54wrky70* double mutant, which was consistent with the up-regulated basal levels of these genes in untreated samples (Figs 1 and 5). There was, however, some variation in the levels of gene expression following the different mock treatments used. Nevertheless, only low/moderate level expression of the genes was found in the wild type, *wrky54*, *wrky70*, *sid2-1* single, *wrky54wrky70sid2-1* triple mutants as well as the *WRKY70* overexpressor line after the two mock treatments (Fig 5). In contrast, after 24 h of *P*. *carotovorum* or *B*. *cinerea* inoculation, these four genes *PDF1*.*2*, *PR3*, *PR4*, *PAD3* were highly induced in the different genotypes (Fig 5), the only exception being the *WRKY70* overexpressor line, which in many cases showed only moderate up-regulation of the genes. The results of gene expression analysis indicated that JA/ET-mediated signaling pathways in *wrky* mutants were not blocked. However, there were no clear differences in pathogen induced gene expression between the different mutant genotypes (Fig 5), thus providing no clear explanation for the observed necrotroph resistance in the double mutant. The enhanced basal expression of the genes in the double mutant might, however, provide partial protection in the early phases of the infection. Furthermore, the results suggest that WRKY54 and WRKY70 together might negatively affect the basal expression of these JA/ET responsive genes.

**Fig 6:**
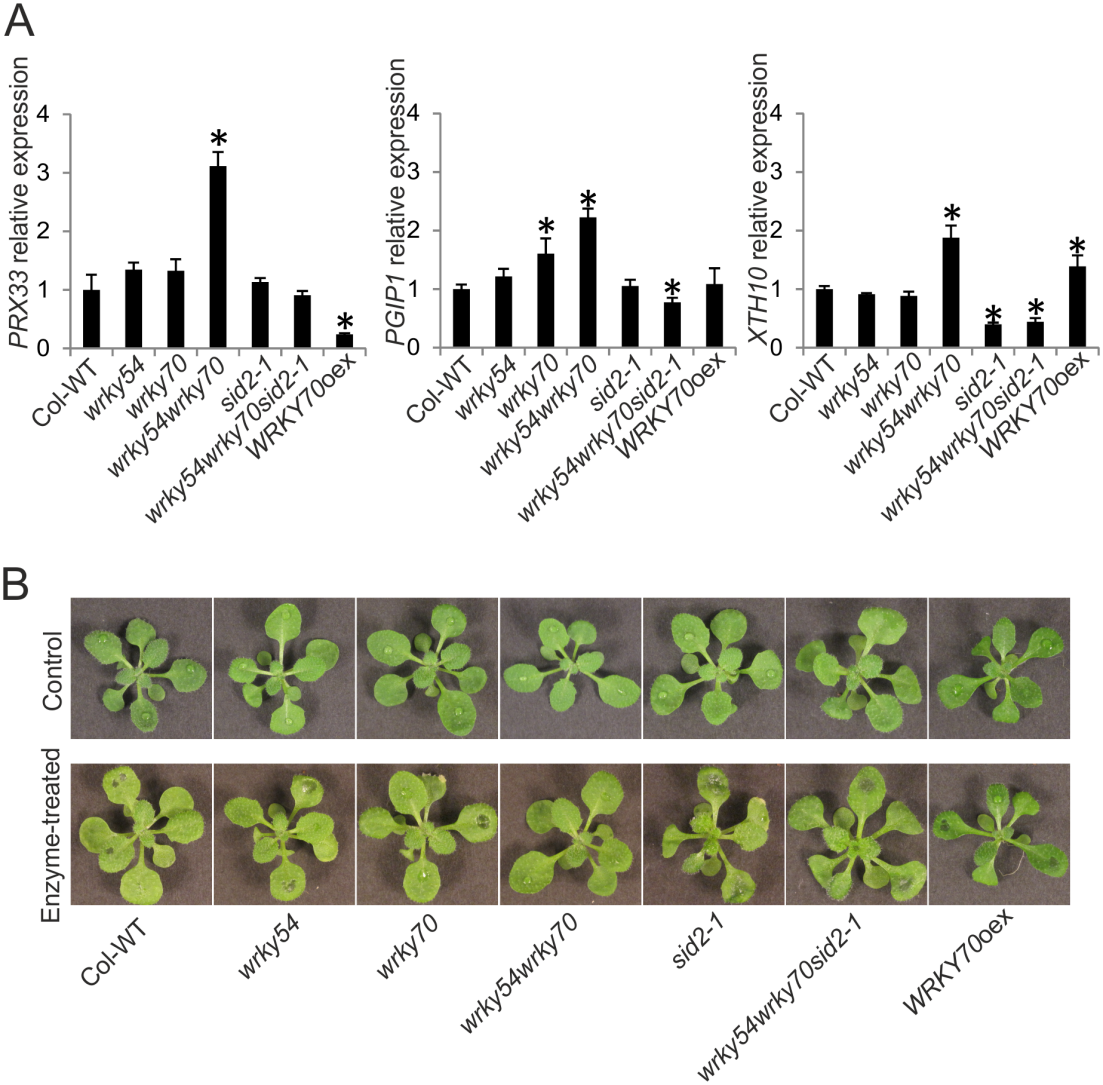
Comparison of cell wall-associated gene expression and cell wall fortification in Arabidopsis wild type (Col-WT), *wrky54*, *wrky70*, *sid2-1* single, *wrky54wrky70* double, *wrky54wrky70sid2-1* triple mutants, as well as *WRKY70* overexpressor line (*WRKY70*oex). (A) Leaves from untreated three-week-old plants were collected. Gene expression was assayed by quantitative reverse transcription-polymerase chain reaction (qRT-PCR). The relative expression of each gene was normalized to that of *ACT2*. Values were obtained from the means ± SD of three technical replicates (*, P<0.01, one-way ANOVA test). Three independent assayed were performed with the similar results. (B) Three-week-old Arabidopsis seedlings were inoculated with cell free supernatants of overnight cultures of *P*. *carotovorum* (pipetting the cell free supernatants to three leaves of each plant). Seventy-two plants of each line in one tray were used in one independent experiment. The representative plants were photographed 6h after inoculation. Three independent experiments were performed with similar results.

In addition to the up-regulation of JA/ET controlled defense-related marker genes in *wrky54wrky70* double mutant, more than 30 cell wall-related genes in GO class 0005618 were also up-regulated in the non-induced double mutant (S1, S2 and S4 Tables). Interestingly, 92% of the genes in this GO class are SA-induced according to genevestigator data [34]. To confirm the array data, three representative genes were chosen from the above GO class 0005618 (S2 and S4 Tables) and their expression was verified by qRT-PCR in unstressed Col-WT, *wrky54*, *wrky70*, *sid2-1* single, *wrky54wrky70* double, *wrky54wrky70sid2-1* triple mutants as well as the *WRKY70* overexpressor line (Fig 6A). One of the genes, *PEROXIDASE 33* (*PRX33*) encoding a cell wall-associated class III peroxidase, was up-regulated in the *wrky54wrky70* double mutant compared to the other lines. Based on the genevestigator data [34], this is a SA-responsive peroxidase and could provide a source of the accumulated H_2_O_2_ in the double mutant. The other two genes *PGIP1* (*POLYGALACTURONASE INHIBITING PROTEIN 1*) and *XTH10* (*XYLOGLUCAN ENDOTRANSGLUCOSYLASE/HYDROLASE 10*) encoding SA-responsive cell wall-modification proteins [34], were slightly up-regulated in the non-induced *wrky54wrky70* double mutant but suppressed in *wrky54wrky70sid2-1* triple mutant, indicating a possible role for SA in cell wall modification (Fig 6A).

The enhanced resistance to the necrotrophic pathogens *P*. *carotovorum* and *B*. *cinerea*, whose virulence strategies mainly rely on CWDEs, and the up-regulation of cell wall-related genes (S4 Table) observed in the *wrky54wrky70* double mutant prompted us to monitor cell wall integrity in the different plant lines (Fig 6B). To achieve this aim, plants were exposed to cell free preparations of *P*. *carotovorum* CWDEs and the extent of leaf maceration monitored. After 6h of drop inoculation by CWDE preparations, the *wrky54wrky70* double mutant showed clearly less macerated leaves compared to the other lines. In contrast, introduction of the *sid2-1* allele resulted in drastically enhanced maceration, highlighting the role of SA also in cell wall fortification. Similarly, also the *WRKY70* overexpressor line showed reduced tolerance to the CWDEs (Fig 6B).

**Fig 7:**
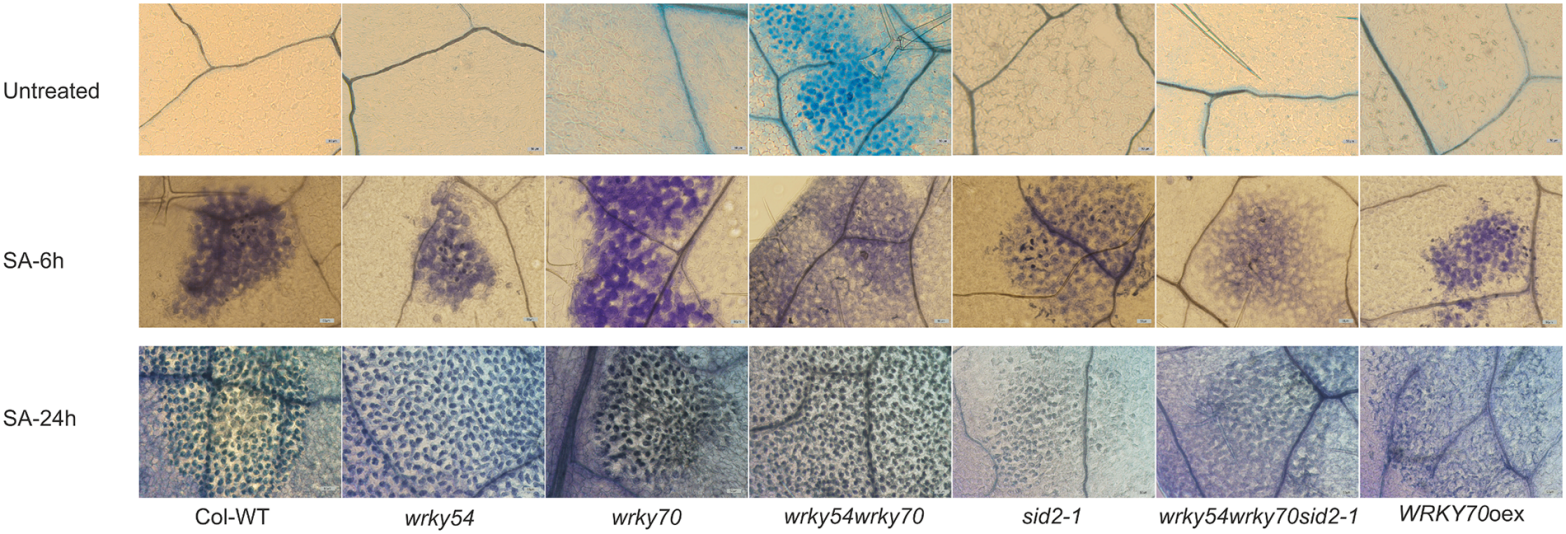
Cell wall modifications (protein cross-linking) in non-treated and SA-treated Arabidopsis wild type (Col-WT), *wrky54*, *wrky70*, *sid2-1* single, *wrky54wrky70* double, *wrky54wrky70sid2-1* triple mutants, as well as *WRKY70* overexpressor line (*WRKY70*oex). Protein cross-linking (dark blue dots) was visualized with coomassie blue staining in non-treated and SA-treated plants. At least three leaves from independent plants of each line at different time points were examined and three independent experiments were performed with similar results. Representative leaves are shown. Scale bar = 50μm.

To visualize possible alterations in the leaf cell walls in the different plant lines, we used coomassie blue staining to detect cross-linking of cell wall proteins (Fig 7). In the analysis of all the genotypes under non-induced and SA-induced conditions, the blue spots indicating protein cross-linking were only visible in epidermal cells of the *wrky54wrky70* double mutant under the non-induced condition (Fig 7), suggesting that cell wall protein cross-linking was more abundant in the *wrky54wrky70* double mutant than in the other lines. However, the blue spots could also be seen in other lines after 6 h of SA induction, and this protein cross-linking was further promoted at the time point of 24 h (Fig 7), indicating the possible role of SA in cell wall fortification.

Taken together these data suggest the enhanced resistance of the double mutant to necrotrophs might involve up-regulated expression of JA/ET responsive genes and is promoted by SA-dependent cell wall fortification manifested for example in protein cross-linking in epidermal cells of the double mutant.

## Discussion

**Fig 8:**
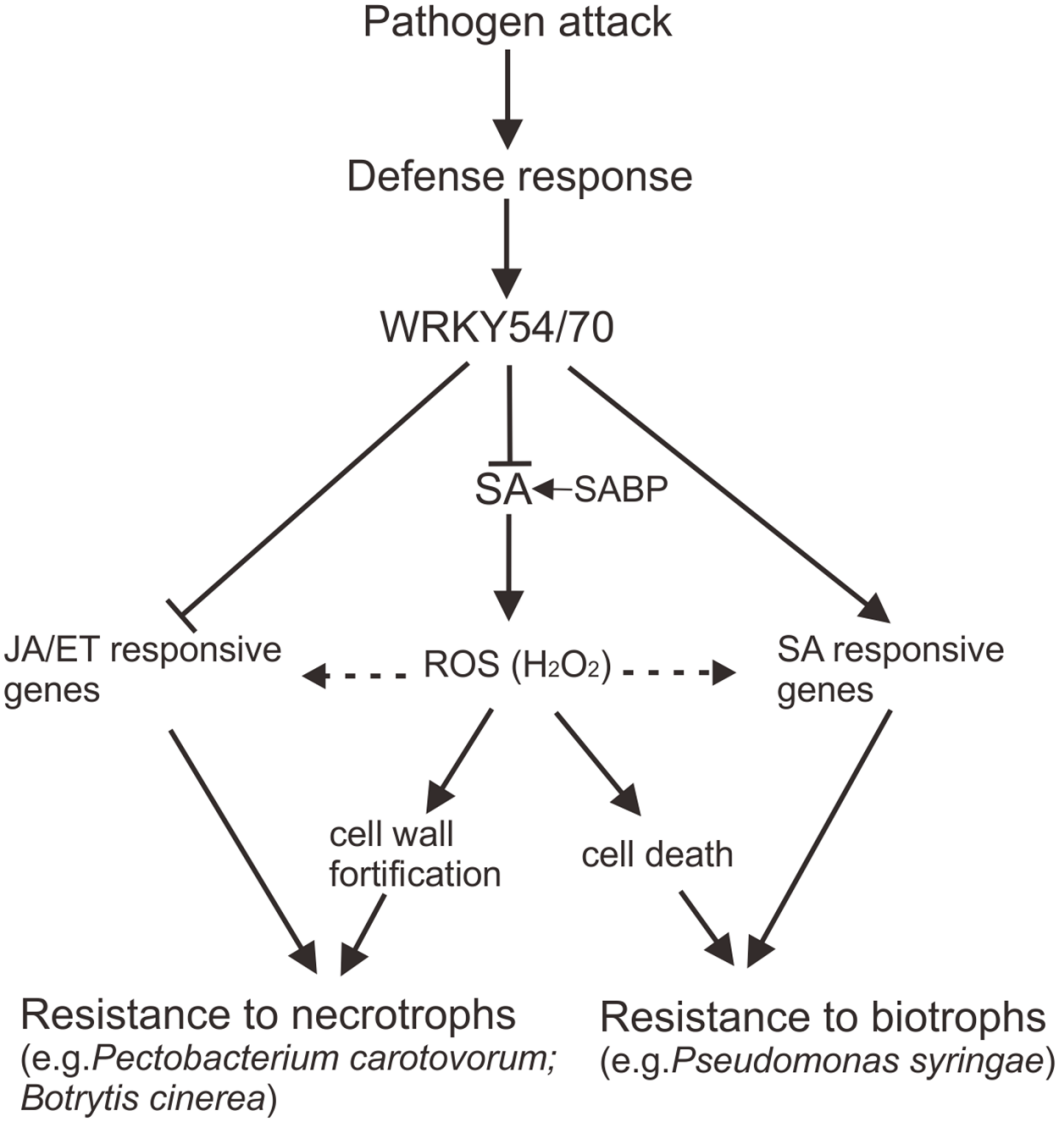
A schematic model of WRKY54- and WRKY70-mediated negative regulation of resistance to necrotrophs. WRKY54 and WRKY70 cooperate as negative regulators of the resistance of Arabidopsis to necrotrophic pathogens. Lack of WRKY54 and WRKY70 results in SA-induced accumulation of H_2_O_2_ leading to cell wall-associated basal antimicrobial defenses. The arrows indicate induction or positive modulation; the blunt-end arrows indicate block or suppression; the dotted line represents promoting or triggering. SA, salicylic acid; JA, jasmonic acid; ET, ethylene; SABP, SA-binding protein; ROS, reactive oxygen species; H_2_O_2_, hydrogen peroxide.

As demonstrated by previous reports, WRKY54 and WRKY70 cooperate as negative regulators of SA biosynthesis and positive regulators of SA-mediated defense signaling in Arabidopsis [8, 21, 22]. In this study, we have provided new insights in the roles of WRKY54 and WRKY70 in cooperatively regulating disease resistance to necrotrophs (Fig 8). Loss of function of both WRKY54 and WRKY70 remarkably enhanced resistance of the corresponding *wrky54wrky70* double mutant to necrotrophic bacterial and fungal pathogens *P*. *carotovorum* and *B*. *cinerea*, respectively (Figs 2 and 3), but not to the hemibiotrophic pathogen *Pst* DC3000 (Fig 4). This is in agreement with previous work by Wang *et al*. [22] who did not observe any increase in resistance in the double mutant to the hemibiotroph *Psm* ES4326. The corresponding single mutants, particularly the *wrky70* mutant, showed slightly increased resistance to necrotrophs. In contrast, the overexpressor of *WRKY70* showed clearly increased resistance to the hemibiotroph *Pst* DC3000 confirming our previous studies [8], but was still sensitive to necrotrophs (Figs 2 and 3). This latter point appears controversial to Li *et al*. [8] who showed that overexpressor of *WRKY70* exhibited somewhat enhanced survival after 5 and 7d of *P*. *carotovorum* infection. This might be explained by the different timing of infection and the different assays used to assess resistance: long term survival in Li *et al*. [8] compared to determining the extent of tissue maceration and bacterial growth in planta 24h post-inoculation in local leaves in the current study. Interestingly, our studies are supported by Li *et al*. [21] who showed that mutants of *wrky70* are more resistant to another fungal necrotroph *Alternaria brassicicola* while *WRKY70* overexpression results in enhanced susceptibility to this pathogen. Conversely, resistance to the fungal biotroph *Erysiphe cichoracearum* was promoted by *WRKY70* overexpression and impaired in *wrky70* mutants [21]. Taken together these data indicate that WRKY54 and WRKY70 cooperate as negative regulators of resistance to necrotrophic pathogens, with WRKY70 having a more prominent role in the process.

WRKY54 and WRKY70 have been shown to cooperate as negative regulators of SA biosynthesis [22], and indeed the *wrky54wrky70* double mutant exhibits substantially increased levels of SA [24]. We could show that this increase in SA was required for the observed resistance to necrotrophs by the introduction of the *sid2-1* allele to the *wrky54wrky70* background (Figs 2 and 3). The *sid2-1* mutant is defective in SA biosynthesis and the resistance phenotype seen in the double mutant was indeed abolished in the *wrky54wrky70sid2-1* triple mutant (Figs 2 and 3). These results suggest that resistance to necrotrophic pathogens could depend on SA-controlled processes although the defenses to necrotrophs are normally considered to be mainly regulated by JA/ET [1, 35, 36]. This notion is also supported by our earlier studies showing that resistance to the bacterial necrotroph *P*. *carotovorum* could be induced by exogenous SA [37].

The elevated SA level in the *wrky54wrky70* double mutant has been demonstrated previously [22, 24], and our microarray data showed that a number of pathogen related genes were up-regulated in the *wrky54wrky70* double mutant under the non-induced conditions (S1 Table). These up-regulated defense-related genes were reduced to the normal wild-type levels in the *wrky54wrky70sid2-1* triple mutant (S1 Table). This indicated that SA plays an important role also for this up-regulated defense gene expression in the *wrky54wrky70* double mutant. Interestingly, in parallel with up-regulation of SA responsive *PR* genes such as *PR1* and *PR2*, genes associated with JA/ET defense signaling, e. g. *PDF1*.*2* and *PAD3* were also up-regulated in the non-treated *wrky54wrky70* double mutant (Fig 1). This is somewhat unexpected as the antagonistic cross talk between SA and JA signaling is widely recognized, with SA mediated signaling often suppressing JA-responsive genes and vice versa [21, 38–43]. However, the antagonistic effect of SA on JA responsive gene expression was not notable when SA signaling was activated before the onset of JA signaling [40]. Therefore, it is possible that the constantly high level of SA in the *wrky54wrky70* double mutant [22, 24] did not suppress the up-regulation of JA/ET responsive genes.

Although SA and JA/ET dependent signaling pathways were both activated as evidenced by up-regulation of both SA- and JA/ET-responsive genes, non-treated *wrky54wrky70* double mutant only showed enhanced resistance to bacterial, *P*.*carotovorum*, and fungal, *B*. *cinerea*, necrotrophs but not to the bacterial hemibiotroph *Pst* DC3000. It appears that some uncharacterized WRKY54 and WRKY70 controlled processes were not activated in the double mutants although most SA-dependent genes were up-regulated. Consequently, the missing processes requiring WRKY54 and WRKY70 might be necessary for development of resistance to biotrophs/hemibiotrophs. In addition, pathogens, like *Pst* DC3000 produce effector proteins, which subvert host immunity and compromise the defense signaling in plants [44]. This might complicate the interpretation of the phenotypes of the *wrky54wrky70* double mutant.

Moreover, JA/ET-mediated signaling could dominate over SA-mediated signaling when necrotrophic pathogens infected the plants. Therefore, synergistic interaction of SA and JA/ET might occur in the pre-alerted state of defense in the non-treated *wrky54wrky70* double mutant, whereas the antagonistic interaction between SA and JA/ET could take place when pathogens invaded the plant. This is in agreement with the notion that in order to fine-tune the defense responses in plants, the appropriate hormone-mediated signaling pathway should be employed while the inappropriate one needs to be shut down accordingly [6].

How is the SA-mediated resistance to necrotrophs executed? As discussed earlier, the up-regulation of defense related genes could only provide a partial answer. A more plausible explanation could be provided by the observed accumulation of ROS in the *wrky54wrky70* double mutant. ROS, such as H_2_O_2_, is usually produced to high levels when plants are attacked by pathogens [45, 46]. Interestingly, increased levels of H_2_O_2_ were found in non-infected *wrky54wrky70* double mutants (Fig 2D), accompanied with up-regulated expression of defense related genes and enhanced resistance to necrotrophs (Figs 1–3), implying that the basal defense in the *wrky54wrky70* double mutant was already preformed. It is likely that the accumulation of H_2_O_2_ was induced by SA (Fig 8). The coordination of SA and H_2_O_2_ has been investigated in previous reports, which demonstrated that SA and H_2_O_2_ formed a positive feedback loop in response to pathogens. SA either acted downstream of elevated H_2_O_2_ or potentially triggered the production of H_2_O_2_, leading to the activation of antimicrobial defenses in the plant [20, 29, 30]. Moreover, SA was also suggested to be involved in the local response to the necrotrophic pathogen, *B*. *cinerea*, in addition to JA/ET [47, 48]. These findings are consistent with our model (Fig 8) indicating that the *wrky54wrky70* double mutant retained high level of SA and consequently an enhanced level of H_2_O_2_ promoting resistance against necrotrophic pathogens.

The outcome of the involvement of ROS in plant-microbe interaction can be varied, depending on the intensity of the ROS signals [17]. High dosage of ROS leads to hypersensitive reaction and induce HR-related cell death, whereas the moderate and balanced level of ROS can trigger the expression of set of defense related genes, production of antimicrobial compounds as well as cell wall fortification through oxidative cross-linking [17, 49–51]. Interestingly, genes encoding cell wall-bound peroxidases and cell wall modification proteins such as *PRX33* and *PGIP1* were up-regulated in the *wrky54wrky70* double mutant (S4 Table and Fig 6A). This suggests that strengthening of preformed defenses against necrotrophs in the *wrky54wrky70* double mutant could be promoted by further accumulation of ROS possibly through the action of cell wall-bound peroxidases (i.e. PRX33 and others) and by other cell wall modifications including protein cross-linking (Fig 7). Most of necrotrophic pathogens rely on disruption of plant cell wall by their CWDEs, which promote maceration [52, 53]. Consequently, up-regulation of *PGIPs* encoding polygalacturonase inhibitor proteins targeting one of the major degradative enzymes would clearly attenuate the virulence of necrotrophs [54, 55]. Therefore, the cell wall in the *wrky54wrky70* double mutant might be fortified and consequently protect plants against maceration by *P*. *carotovorum* and *B*. *cinerea*. Nevertheless, we could not ignore that the expression levels of *PRX33*, *PGIP1* and *XTH10* in *wrky54wrky70* double mutant were only slightly higher than the other lines, whereas the protein cross-linking results indicated very clear difference between *wrky54wrky70* double mutant and the other lines (Figs 6 and 7). This suggested that the fortified cell wall in *wrky54wrky70* double mutant might be caused not only by the up-regulation of these genes, but also some other genes involved in this process. Hence, future research will focus on the identification of SA-responsive target genes mediating the cell wall fortification.

In addition to the role of ROS in plant cell wall modification, ROS, such as H_2_O_2_, are widely considered as signaling molecules triggering cell death. As indicated earlier, cell death is beneficial for plant resistance to biotrophic pathogens but can promote the virulence of necrotrophs [31]. For example, the aggressiveness of *B*. *cinerea* and *Sclerotinia sclerotiorum* is highly dependent on the level of ROS, and *B*. *cinerea* can even generate ROS itself to promote successful infection [46, 56]. Interestingly, the *wrky54wrky70* double mutant did not show any H_2_O_2_-induced cell death symptoms under the control condition (Fig 3C), suggesting that the dosage of H_2_O_2_ accumulated in the double mutant might not be high enough to induce cell death, but only trigger the cell wall-mediated resistance to necrotrophs. In contrast, overexpression of *WRKY70* enhanced cell death after infection although it did not show cell death before infection (Fig 3C). This is in concert with the rapid activation of oxidative burst after pathogen infection in *WRKY70* overexpressor revealed by DAB staining, and results in enhanced resistance to *Pst* DC3000 but not to *P*. *carotovorum* and *B*. *cinerea*. Therefore, although ROS were accumulated in both the *wrky54wrky70* double mutant and the *WRKY70* overexpressor, the outcome of defense to pathogens was different. We propose (Fig 8) that the early accumulation of H_2_O_2_ in the *wrky54wrky70* double mutant is moderate and results in cell wall-associated antimicrobial defenses; whereas the high dosage of H_2_O_2_ locally accumulated in *WRKY70* overexpressor leads to rapid HR induced cell death and resistance to the biotrophic pathogens. This model is further supported by examples from tomato, where early accumulation of ROS enhanced the resistance of the abscisic acid (ABA)-deficient mutant *sitiens* to *B*. *cinerea* due to the fortification of epidermal cell wall through protein cross-linking [57] as well as from Arabidopsis, where the *ocp3* (*overexpressor of cationic peroxidase 3*) mutant showed reduced susceptibility to *B*. *cinerea* and *Plectosphaerella cucumerina* as a consequence of increased basal level of H_2_O_2_ in the mutant [30]. In conclusion (Fig 8), we suggest that WRKY54 and WRKY70 cooperate in controlling ROS homeostasis in the cell as negative regulators of SA biosynthesis. ROS possibly generated by apoplastic peroxidases in turn contribute to pathogen defense in distinct ways depending on the time of infection, type of pathogen and intensity of the signals.

## Supporting information

S1 Fig Oligogalacturonide (OG)-induced callose deposition detected by aniline blue staining in Arabidopsis wild type (Col-WT), *wrky54*, *wrky70*, *sid2-1* single, *wrky54wrky70* double, *wrky54wrky70sid2-1* triple mutants, as well as *WRKY70* overexpressor line (*WRKY70*oex). The solution containing 100μg/ml OG was sprayed to 3-week-old in vitro plants and the plants were incubated at high humidity for 24h. Water was used as control. At least three leaves from independent plants of each line were harvested and stained for callose. Representative leaves are shown. The experiment was repeated at least two times with similar results. Scale bar = 50μm.
(TIF)

S1 Table The genes up-regulated (log_2_FC≥1.5) in the *wrky54wrky70* double mutant compared to wild-type plants under non-treated conditions, the basal expression levels of these genes in *sid2-1* single and *wrky54wrky70sid2-1* triple mutants are also indicated in the table. (XLS)

S2 Table The significant gene ontology (GO) terms for the genes in S1 Table. (XLS)

S3 Table The primers used in quantitative reverse transcription-polymerase chain reaction (qRT-PCR). (XLS)

S4 Table Up-regulation of cell wall-associated genes (log_2_FC≥1.5) in the *wrky54wrky70* double mutant compared to wild-type plants under non-induced conditions. (XLS)
