# Supplementary figures and images for: WRKY70 and its homolog WRKY54 negatively modulate the cell wall-associated defenses to necrotrophic pathogens in Arabidopsis
Source: PLoS One. 2017 Aug 24;12(8):e0183731. doi: 10.1371/journal.pone.0183731 (PMC5570282; doi:10.1371/journal.pone.0183731)

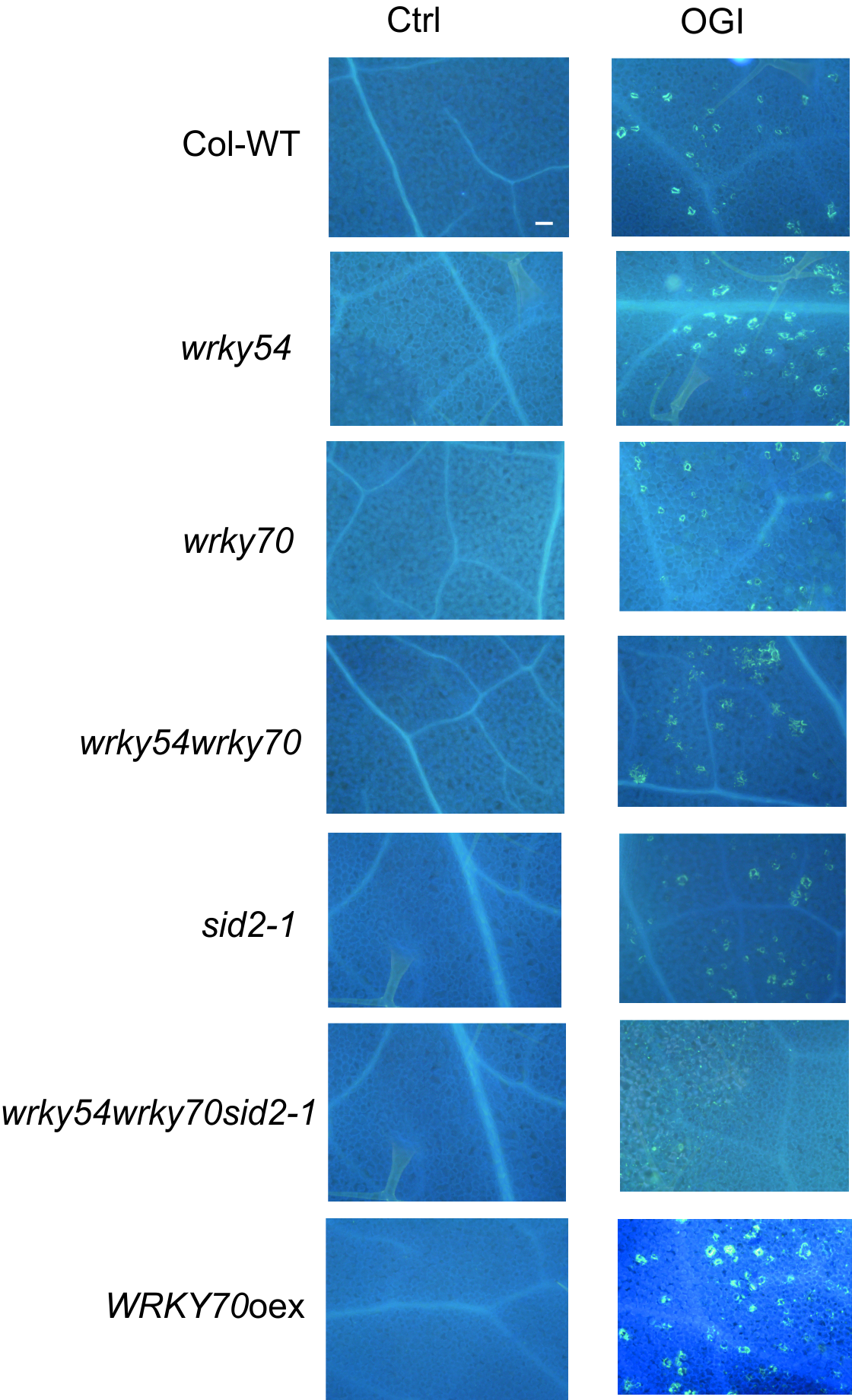

Supplement: S1 Fig — The solution containing 100μg/ml OG was sprayed to 3-week-old in vitro plants and the plants were incubated at high humidity for 24h. Water was used as control. At least three leaves from independent plants of each line were harvested and stained for callose. Representative leaves are shown. The experiment was repeated at least two times with similar results. Scale bar = 50μm. (TIF) [file pone.0183731.s001.tif]
